# Irreversible impact of chronic hepatitis C virus infection on human natural killer cell diversity

**DOI:** 10.15698/cst2018.07.150

**Published:** 2018-07-25

**Authors:** Benedikt Strunz, Julia Hengst, Heiner Wedemeyer, Niklas K. Björkström

**Affiliations:** 1 Center for Infectious Medicine, Department of Medicine Huddinge, Karolinska Institutet, Karolinska University Hospital Huddinge, 14186 Stockholm, Sweden.; 2 Department of Gastroenterology, Hepatology and Endocrinology, Hannover Medical School, 30625 Hannover, Germany.; 3 Department of Gastroenterology and Hepatology, Essen University Hospital, 45147 Essen, Germany.; 4 German Center for Infection Research (DZIF); partner site Hannover-Braunschweig, Medical School Hannover, 30625 Hannover and Helmholtz Center for Infection Research, 38124 Braunschweig.

**Keywords:** Hepatitis C virus infection, direct-acting antiviral therapy, natural killer cells

## Abstract

Diversity is crucial for the immune system to efficiently combat infections. Natural killer (NK) cells are innate cytotoxic lymphocytes that contribute to the control of viral infections. NK cells were for long thought to be a homogeneous population of cells. However, recent work has instead revealed NK cells to represent a highly diverse population of immune cells where a vast number of subpopulations with distinct characteristics exist across tissues. However, the degree to which a chronic viral infection affects NK cell diversity remains elusive. Hepatitis C virus (HCV) is effective in establishing chronic infection in humans. During the last years, new direct-acting antiviral drugs (DAA) have revolutionized treatment of chronic hepatitis C, enabling rapid cure in the majority of patients. This allows us to study the influence of a chronic viral infection and its subsequent elimination on the NK cell compartment with a focus on NK cell diversity. In our recent study (Nat Commun, 9:2275), we show that chronic HCV infection irreversibly impacts human NK cell repertoire diversity.

Due to the fact that infection with HCV nearly always becomes chronic, we first investigated the imprint of the persisting infection on the NK cell compartment. To overcome shortcomings of prior studies, where typically a limited number of phenotypic parameters had been assessed, we applied stochastic neighbor embedding (SNE) analysis to complex flow cytometry data. This analysis revealed that the NK cell receptor repertoire showed distinct patterns in HCV patients as compared to healthy controls. The phenotype was primarily driven by reduced expression levels of activating receptors in the patients. Interestingly, we also noted a high spread of the expressed markers in-between the HCV infected patients while healthy controls clustered closely together. Hence, NK cells from HCV-infected patients displayed a greater variation in how surface receptors were expressed.

Next, we aimed to grasp the altered expression variation on the inter-individual level. Therefore, we implemented donor-to-donor expression variation (DEV) as a novel immune metric that more globally estimates immune population variation between individuals. We also assessed intra-individual variation within the NK cell compartment of chronic HCV patients as compared to healthy controls. To this end, inverse Simpson diversity index (SDI) was employed. SDI is a measure of population diversity originating from ecology, which has previous been used to study NK cell receptor repertoire diversity. We based our SDI-assessment on known NK cell differentiation markers. Taken together, with these measurements, it was possible to determine both inter- and intra-individual NK cell diversity and to estimate the impact of chronic HCV on the measures.

DEV and SDI were first validated on a large cohort of 202 healthy controls in relation to CMV status and secondly in comparing peripheral blood and liver samples. As expected, the inter-individual diversity, DEV, was increased for NKG2C and KIR on NK cells in CMV-positive individuals. These two receptors are often found on CMV-driven adaptive-like NK cell expansions. On intrahepatic NK cells, CD69 had increased DEV as compared to peripheral blood. This was also expected since CD69 rarely is expressed on circulating NK cells but found on liver-resident NK cells. Studying the intra-individual diversity (SDI), we detected significantly lower SDI for NK cells in CMV-positive as compared to CMV-negative individuals. Again, this was anticipated since the CMV-driven adaptive-like NK cell population has a fairly fixed surface receptor phenotype. Due to the specific phenotype of intrahepatic NK cells, we found reduced SDI compared to peripheral blood NK cells in this setting as well. Taken together, these analyses validated DEV and SDI as relevant immune metrics to gauge global NK cell diversity.

Next, we applied the DEV and SDI measurements to the chronic HCV cohort. The previously observed higher spread in the expression level of individual NK cell receptors among the patients led to an elevated DEV as compared to healthy controls (**Figure 1**). Of note, increased DEV was associated with severity of liver fibrosis. Strikingly, chronic HCV infection furthermore caused a significant reduction of NK cell SDI (**Figure 1**). This was independent from CMV serostatus and other clinical parameters such as age, gender, stage of liver fibrosis, and treatment outcome. Distinct from these differences in NK cell receptor repertoire diversity, examination of the NK cells’ ability to react by natural cytotoxicity, respond to cytokine stimulation, or mediate antibody-dependent cell-mediated cytotoxicity revealed no major differences between the chronic HCV patients and healthy controls. Yet, grouping the patients according to their SDI values, into either normal (similar SDI values as healthy controls) or reduced SDI, revealed that patients with reduced SDI displayed impaired cytokine production upon K562 stimulation.

**Figure 1:**
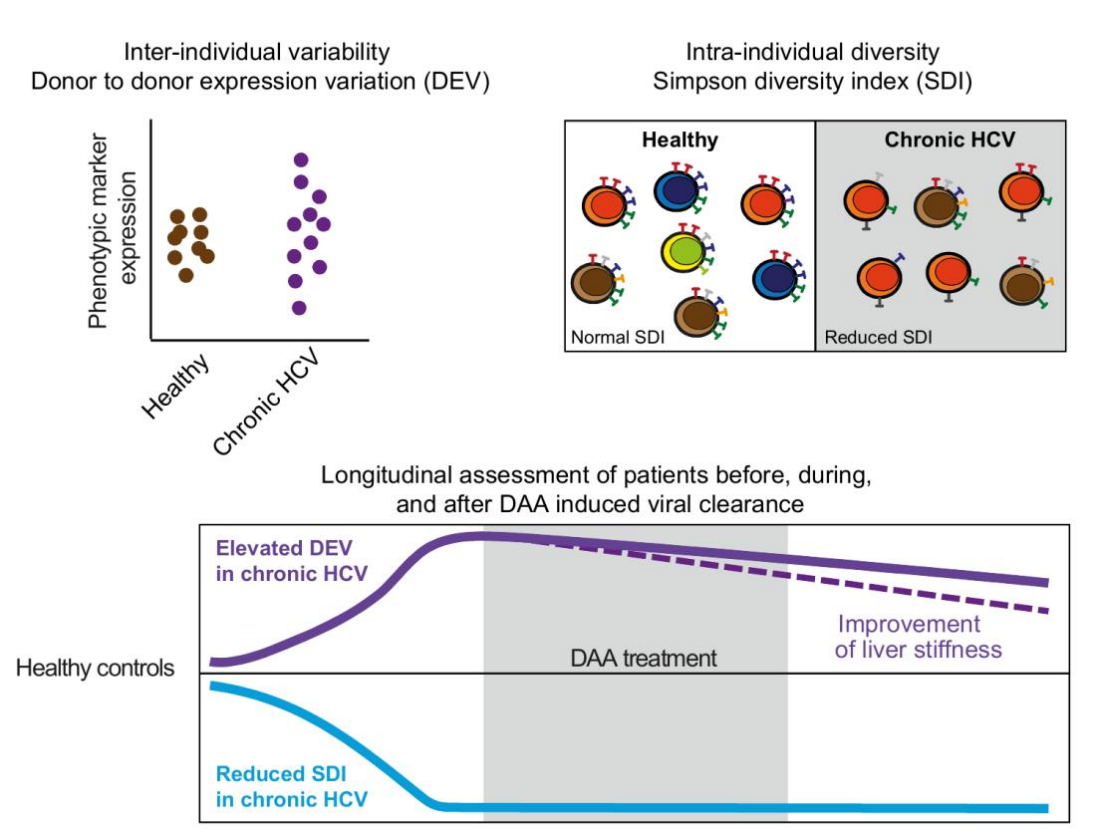
FIGURE 1: Imprint on NK cell diversity by chronic HCV infection. Schematic illustration on how chronic HCV infection affects NK cell inter-individual variability evaluated determining donor-to-donor expression variation (DEV) (top left panel). Assessment of intra-individual diversity within the NK cell population using the Simpson diversity index (SDI) in healthy controls and patients with chronic HCV (top right panel). Longitudinal characterization of DEV and SDI in chronic HCV infection, before, during, and after viral clearance using direct-acting antivirals (DAA) (lower panel).

Finally, patients were followed longitudinally during DAA treatment and up to 48 weeks after treatment completion. In this setting, we addressed if the observed impact of chronic HCV on NK cell diversity normalized after clearance of virus, or if the alterations were irreversible. SNE analysis comparing the NK cell phenotype at baseline and follow-up time-points revealed only few signs of normalization during therapy and after viral clearance. This involved reduction of Granzyme B expression and increase of NKG2D expression levels. Yet, expression of the majority of the examined markers remained unaltered. In line with this, NK cell SDI stayed reduced following viral elimination as well. Thus, the imprint on the intra-individual NK cell diversity appeared to be long-lasting. In contrast, the elevated DEV levels observed during chronic HCV infection decreased one year after successful viral clearance for many of the parameters. This could be attributed to the improvement of liver inflammation and reduced liver stiffness.

In conclusion, our study was one of the first studies to examine the influence of a chronic infection on NK cell receptor repertoire diversity and we also studied this at very late time points after viral clearance. With new immune metrics gauging inter- and intra-individual NK cell diversity, we could identify a previously unrecognized imprint on the NK cell compartment caused by the virus. This was evident by elevated DEV, associated with the severity of liver fibrosis, as well as by reduced SDI in the HCV patients as compared to healthy controls. DEV normalized in parallel with improvement of liver stiffness upon viral clearance. In contrast, no reinvigoration of NK cell SDI was observed even up to one year after successful viral clearance. Thus, a chronic infection is able to leave a permanent imprint on the composition of human NK cells, an imprint that might persist for years after the pathogen has been successfully eliminated.

